# Ultrastrong ductile and stable high-entropy alloys at small scales

**DOI:** 10.1038/ncomms8748

**Published:** 2015-07-10

**Authors:** Yu Zou, Huan Ma, Ralph Spolenak

**Affiliations:** 1 Laboratory for Nanometallurgy, Department of Materials, ETH Zurich, Vladimir-Prelog-Weg 5, CH-8093 Zurich, Switzerland.

## Abstract

Refractory high-entropy alloys (HEAs) are a class of emerging multi-component alloys, showing superior mechanical properties at elevated temperatures and being technologically interesting. However, they are generally brittle at room temperature, fail by cracking at low compressive strains and suffer from limited formability. Here we report a strategy for the fabrication of refractory HEA thin films and small-sized pillars that consist of strongly textured, columnar and nanometre-sized grains. Such HEA pillars exhibit extraordinarily high yield strengths of ∼10 GPa—among the highest reported strengths in micro-/nano-pillar compression and one order of magnitude higher than that of its bulk form—and their ductility is considerably improved (compressive plastic strains over 30%). Additionally, we demonstrate that such HEA films show substantially enhanced stability for high-temperature, long-duration conditions (at 1,100 °C for 3 days). Small-scale HEAs combining these properties represent a new class of materials in small-dimension devices potentially for high-stress and high-temperature applications.

## Abstract (web summary)

Refractory high-entropy alloys show promising mechanical properties at elevated temperatures, but are generally brittle at room temperature. Here, the authors observe an improved ductility and yield strength in high-entropy alloy micropillars consisting of nanometre-sized grains that also exhibit excellent thermal stability.

## Graphical abstract

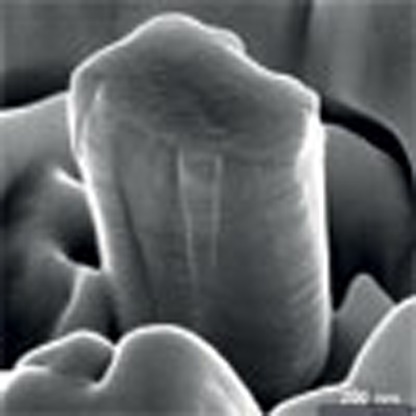

Developing high-strength, ductile and thermally stable materials is highly desirable for both scientific interests and critical applications^1^,^2^,^3^. Alloying has been explored as a means to strengthen metals since the Bronze Age. Conventionally, one principle element serves as the matrix material and solute atoms change local stress fields to impede dislocation motion and strengthen the material, although it usually compromises ductility. Over the past few years, a new concept of alloys—HEAs, or equiatomic multi-component alloys—has attracted great attention^4^,^5^. Such alloys usually consist of four or more elements with equimolar or near-equimolar ratios, form a simple single solid-solution-like phase and show a variety of interesting and unusual properties^6^,^7^. Among them refractory HEAs are made of refractory elements and implemented for high-temperature applications. For example, a body-centred cubic (bcc)-structured NbMoTaW HEA subjected to uniaxial compression at 1,600 °C attain a yield strength of 400 MPa and high heat-softening resistance^8^,^9^. However, all the refractory HEAs reported to date have been prepared using vacuum arc-melting technique and a vast majority of them suffer from low ductility at room temperature^9^,^10^,^11^: rendering them very difficult to process and unsuitable for usage.

The ductility and strength of a material can be also controlled by scaling, that is sample and microstructural sizes^12^,^13^. On the one hand, benefiting from higher surface-to-volume ratios and easier stress relaxation, cracking becomes more difficult in small-sized materials—the good deformability could be attained—even in conspicuous classes of brittle materials^14^,^15^,^16^. On the other hand, materials may attain significantly increased strengths by reducing their dimensions due to a limited scale of dislocation sources^17^,^18^,^19^,^20^,^21^. To achieve even higher strengths, a popular methodology is to include grain or interphase boundaries in micro- or nano-pillars, namely nanocrystalline or nanolaminate pillars, respectively. These nanostructured pillars can reach yield strengths of a few gigapascals^22^,^23^,^24^,^25^, but their main drawback is that their microstructures are generally unstable: grains grow rapidly even at low temperatures, consequently their strengths decrease considerably. To stabilize nanocrystalline structures, a few effective means have been introduced to suppress grain growth, such as alloying^26^,^27^ and introducing texture^28^.

So far promising HEAs have been mostly studied in their bulk forms, but small-dimension HEAs have received much less attention. As demands for micro- and nano-scale devices for high-temperature and harsh-environment applications increase, the fabrication and investigation currently popular HEAs at small sizes become more and more interesting. Now, the following question arises: what alloying and scaling conditions lead to the strongest both ductile and stable materials? Our strategy is to use the sample size and grain size as design parameters in a prototype refractory HEA, NbMoTaW alloy, to combine the benefits of alloying and scaling. Here, we show that fine-scale HEA films and pillars consisting of strongly textured, nanometre-sized and columnar grains exhibit ultrahigh strength, improved ductility and excellent thermal stability.

## Results

### Nanostructured HEA films and pillars

**Figure 1:**
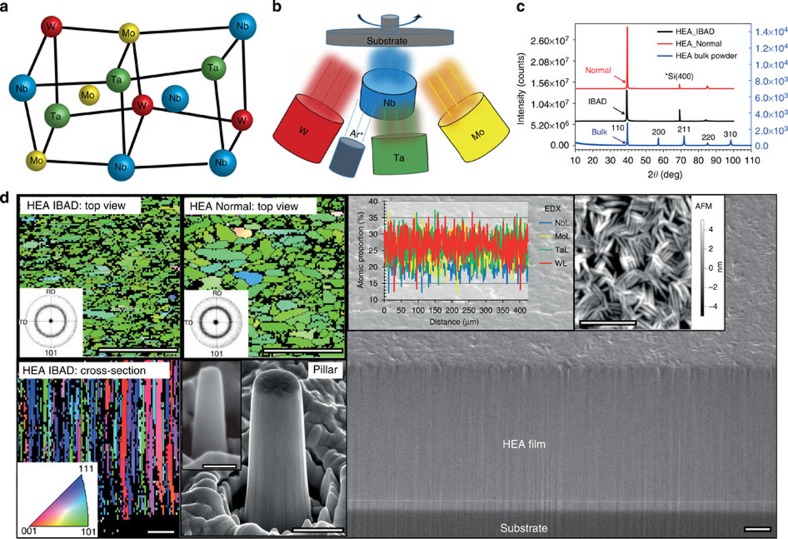
Fabrication and characterization of NbMoTaW HEA films and pillars. (**a**) Schematic representation of an ideal lattice structure of a bcc NbMoTaW HEA. (**b**) Schematic illustration of the d.c. magnetron co-sputtering system used to synthesize HEA thin films, in the conditions with and without Ar^+^ ion beam-assisted deposition: IBAD and Normal, respectively. (**c**) Powder X-ray diffraction patterns (Cu Kα1) of the NbMoTaW HEA films, compared with that of its bulk powder^11^, indicating a single bcc phase. (**d**) A SEM image of the typical cross-section of as-deposited IBAD HEA films. The inserted EBSD maps show columnar grains through the whole thickness of the films with a (110) out-of-plane texture and an average grain size of ∼70 and ∼150 nm for the IBAD and Normal conditions, respectively. The EDX analysis indicates that the four elements are homogenously distributed in a large length scale. The roughness of the top surface measured by AFM is about 5 nm. Two representative FIB-milled pillars (diameters of ∼500 and ∼100 nm) are shown in the insert at the bottom. Scale bars, 500 nm, except the one for ∼100 nm pillar is 100 nm.

We used d.c. magnetron co-sputtering technique to deposit HEA films, as schematically illustrated in Fig. 1a,b (also see the experimental setup in Supplementary Fig. 1). Ion beam-assisted deposition (IBAD) method^29^ was also applied to reduce deposition rate and decrease grain size. For simplicity, the method without using the ion gun is named as ‘Normal' as opposed to ‘IBAD'. Using the co-sputtering method, we produced 3-μm thick films that show good bonding with substrates and smooth surfaces (Fig. 1d). Electron backscatter diffraction (EBSD) orientation maps (insets in Fig. 1d) show that the films consist of strongly (110) textured columnar grains through the whole thickness of both IBAD and Normal-deposited films. The films deposited under the IBAD condition exhibit smaller grain sizes than those produced under the Normal condition, with an average grain size of ∼70 and ∼150 nm, respectively. The energy-dispersive X-ray spectroscopy (EDX) analysis reveals that the atomic composition varies by ∼5% and the overall value varies within 10%, which is comparable to the previously reported bulk NbMoTaW HEAs^8^,^11^. The X-ray diffraction patterns indicate a single-phase bcc structure in the as-deposited films, which also matches the bulk HEA in literature^8^,^11^. The results in Fig. 1 confirm that the co-sputtered films are made of the same alloy as those bulk forms produced by arc melting.

### Micro-mechanical testing of small-scale HEA pillars

**Figure 2:**
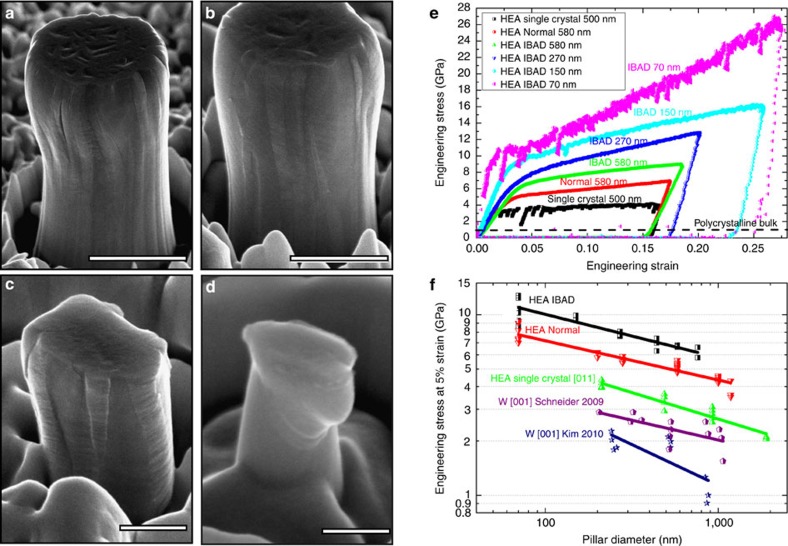
Compression results for the pillars prepared from the HEA films. (**a**–**d**) SEM images of typical as-deformed HEA pillars (IBAD) with the diameter (*D*) ranging from ∼1 μm to 100 nm. (**e**) Representative stress–strain curves of the IBAD HEA pillars, showing a size-dependent strength. (**f**) A comparison of the strength–size relationships for the columnar-structured HEA pillars, single-crystal HEA (using the bulk specimen^11^) and W pillars^20^,^21^. The results for the Normal HEA pillars are similar to the IBAD HEA pillars (Supplementary Figs 2 and 3). Scale bars, 1 μm (**a**), 500 nm (**b**), 200 nm (**c**) and 100 nm (**d**).

Focused Ga ion beams (FIB) were used to mill fine-scale pillars out of the obtained HEA films and microcompression tests were carried out using a nanoindenter. After compression a fraction of large pillars, above 1 μm in diameter, experience cracking at the top parts and cracks propagate along grain boundaries, showing intergranular fracture behaviour, but it only occurs under strains larger than ∼20% (Fig. 2a). The smaller pillars (Fig. 2b–d) exhibit more uniform deformation without any cracking, even at above 30% compressive strain, suggesting that the compressive ductility is significantly improved. Furthermore, the columnar-structured HEA pillars exhibit very high yield and flow strengths. A 580-nm Normal HEA pillar shows a yield strength of ∼5 GPa and a 580-nm IBAD HEA pillar exhibits a yield strength of ∼6.5 GPa (Fig. 2e), which is almost twice of that of the single-crystal HEA pillar with the same diameter and orientation (Supplementary Fig. 4) and six times of that of the bulk HEA. Astonishingly, we find that the smallest IBAD HEA pillars (∼70–100 nm in diameter) exhibit remarkably high yield strengths of ∼8–10 GPa. To the best of our knowledge, such HEA pillars exhibit a strengthening figure of merit that is among the strongest pillars reported so far—for example, nanocrystalline Ni–W pillar, ∼1 GPa (ref. 24); nanocrystalline Zr pillar, ∼4 GPa (ref. 30); nanolaminate Cu/Nb, ∼2 GPa (ref. 25); Si, ∼6 GPa (ref. 14); GaN, ∼8 GPa (ref. 31); CrAlN/Si_3_N_4_, ∼16 GPa (ref. 32) and Zn-based metallic glasses^15^, ∼2 GPa—and are in the same strength level of the defect-free Mo-alloy columns produced from etching NiAl–Mo eutectic compounds^33^ and about half of that of pure W whiskers^34^, still our HEA pillars exhibit much better ductility. Such HEA pillars also show a size-dependent strength, as presented by the relationship between the flow stress at 5% strain, *σ*_0.05_, versus the pillar diameter, *D* (Fig. 2f). Our IBAD HEA pillars exhibit the highest strength levels, ∼5–7 times higher than that of single-crystal W pillars, and the lowest size dependence, a log–log slope of −0.2.

### Thermal stability of the HEA thin films

**Figure 3:**
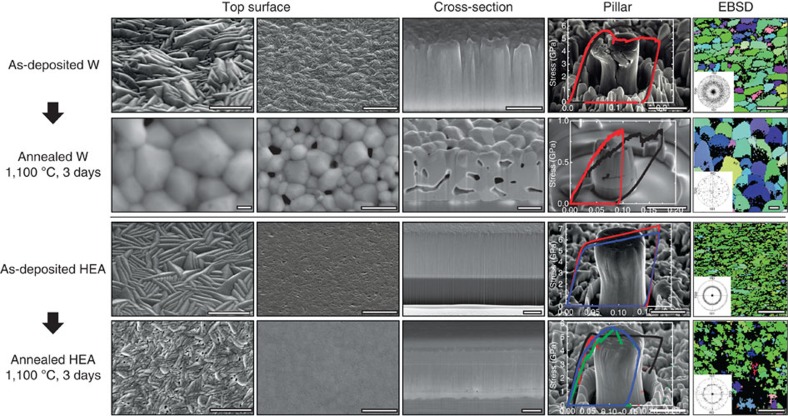
Pre- and post-annealing structures of the W and HEA films after 3 days at 1,100 °C. The top surfaces, cross-sections and grain structures indicate significant grain coarsening, pore formation and morphology change in tungsten films upon annealing, but the difference of microstructure and strengths of the HEA films before and after annealing is minor under the same deposition and annealing conditions. Scale bars, 200 nm (the first column, large magnifications of top surfaces); 300 nm (the last column, EBSD maps); 1 μm (the other images).

In addition to ultrahigh strength and improved ductility, we also demonstrate that such HEA films are substantially more stable after high-temperature, long-duration annealing compared with the pure W films that were prepared using the same experimental conditions. After 3 days' annealing at 1,100 °C in an argon atmosphere the pure W film shows obvious structural instability: the morphology of the top surface changes from needle-like shapes to equiaxed-crystal structures; a large quantity of micrometre-sized pores are formed through the whole thickness; the grain size is significantly increased from ∼100 to 300 nm to a few micrometres, as shown in Fig. 3. In contrast to the W films, the post-annealed HEA film retains uniform needle-like morphology on the top surface without obvious grain growth, and few pores have been found through the entire cross-section of the films. With regards to mechanical properties, the HEA pillars exhibit much higher strength and better ductility than the W pillars before and after annealing (see a deformed W pillar before annealing in Supplementary Fig. 5). The formation of micropores and the growth of grains may dramatically reduce the mechanical performance of the W films and pillars, while the post-annealed HEA pillar (diameter of ∼1 μm) can still sustain a high yield strength of ∼5 GPa, which is nearly the same as that of the pre-annealed HEA pillar.

## Discussion

**Figure 4:**
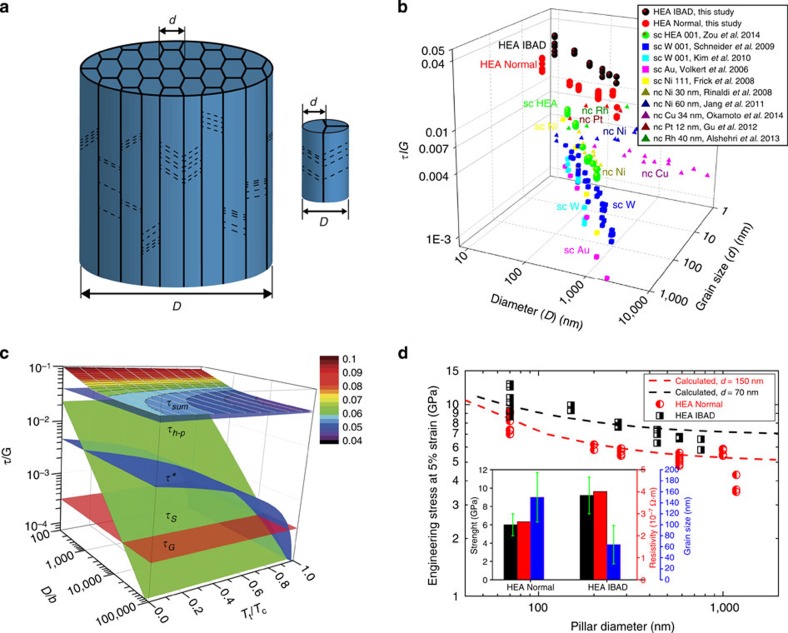
Size-dependent strength in column-structured pillars. (**a**) Schematic illustrations of a big pillar and a small one, with *D* the pillar diameter and *d* the grain size. (**b**) A three-dimensional (3D) graph shows the relation of normalized resolved shear strength (*τ/G*) versus (*D* versus *d*) for the HEA pillars in this study, single-crystalline (sc) HEA^11^, bcc W^20^,^21^, fcc Au^18^, Ni^19^ pillars and nanocrystalline (nc) Cu^22^, Ni^23^, Ni–W^24^, Pt^44^ and Rh^45^ pillars, with *τ* resolved shear strength and *G* the corresponding shear modulus. The Schmid factors of 0.417 and 0.5 are used for the bcc HEA pillar and the nc pillars in the available literature data, respectively. The sample sizes of sc pillars can be regarded as their grain sizes as well. (**c**) A 3D illustration of size and temperature dependence of the strengths for the HEA pillars ((*τ/G*) versus (*D/b*) versus (*T*_*t*_*/T*_*c*_)), as described in equation (1). (**d**) A comparison between the calculated strengths using equation (1) and the experimental values for the HEA pillars. The inset compares pillar strengths, average grain sizes and resistivity of Normal and IBAD HEA films.

In analogy to bundled bamboos, our column-structured HEA pillars actually consist of a set of strongly fibre-textured nanometre-sized grains, schematically illustrated in Fig. 4a. As a comparison of the normalized strengths (resolved shear strength (*τ*) over corresponding shear modulus (*G*)), the IBAD HEA pillars exhibit the highest values (∼0.02–0.05) among the typical single-crystalline pillars and nanocrystalline pillars (Fig. 4b). To understand the ultrahigh strength of the HEA pillar, we propose a simple classical analysis on the resolved flow strength of the pillar (*τ*_sum_), which is contributed by lattice friction (τ*), Taylor hardening (*τ*_G_) and source-controlled strength (*τ*_S_) and grain-boundary strengthening (*τ*_h–p_), simply expressed as (adapted from refs 11, 35, 36):


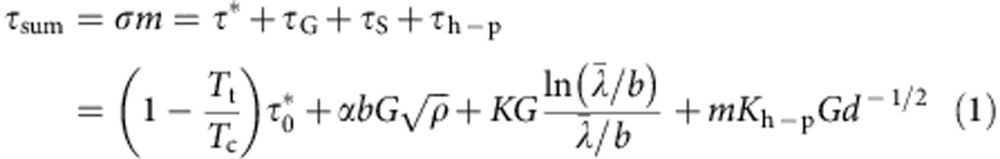


Where *σ* flow stress, *m* Schmid factor, *T*_t_ test temperature, *T*_c_ critical temperature (above *T*_c_ flow stress becomes insensitive to test temperature), 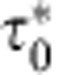 the Peierls stress, *α* a constant falling in the range 0.1–1.0, *b* the Burgers vector, *G* shear modulus, *ρ* dislocation density, *K* source-strengthening constant in the order of 0.1, 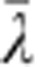 average source length and *K*_h−p_ Hall–Petch constant. Figure 4c presents a three-dimensional illustration of the additivity of different strengthening mechanisms in a size range of 10^2^–10^5^*b* and a temperature range of 0–*T*_c_. To make a comparison with the experimental data, we choose the following parameters for the HEA pillars to give the best estimation: *m* 0.417 (the most probable slip systems [111]
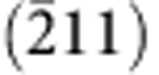
 with [011] loading direction), *T*_t_ 300 K, *T*_c_ 1,050 K (ref. 9), 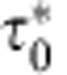 446 MPa (the average value for Nb, Ta, Mo and W)^11^, *α* 0.5, *b* 2.799 Å (ref. 11), *ρ* 5.0 × 10^12^ m^−2^, *K* 0.5, 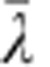 is proportional to the pillar dimension (as a function the sample volume^37^) which can be simply represented by *D*, *K*_h−p_ 1.7 GPa μm^1/2^ (for bulk Mo)^38^. The calculated values are in good agreement with the experimental data (Fig. 4d), implying that the four possible strengthening mechanisms could work simultaneously in the nanostructured HEA pillars. It should be also mentioned that the smallest pillars (∼70–100 nm in diameter) show obviously higher scattering levels in strength compared with the larger pillars. This large scattering could attribute to the inhomogeneous distribution of grain boundaries in these small pillars. In addition, the higher strengths of the IBAD pillars than those of the Normal pillars could be mainly attributed to their finer grain sizes. The higher point defect density in the IBAD pillars (as measured by electrical resistivity shown in the inset of Fig. 4d) could influence the strength as well, but its contribution is deemed to be small.

It is also instructive to look at a thought experiment regarding strength and fracture. One could consider comparing this bundled-bamboo structure to a discrete array of single-crystalline pillars of identical dimension as the grain size. With regards to strength these single-crystalline pillars would be close to theoretical strength, provided they are defect-free. If some of them are not, the overall strength of the array would be slightly reduced and only decrease significantly if the overall number of defects were increased by increasing the number of pillars, that is, increasing the diameter of the whole pillar. In the bundled-bamboo structure itself, yielding of a single grain will result in stress concentrations at the boundaries, activating dislocation sources in the adjacent grains^39^ and, therefore, yielding in those as well leading to a reduced overall yield strength compared with the theoretical one. This is an alternative explanation of the size-dependent strength in the HEA pillars. With regards to fracture the single-crystalline pillars, each columnar grain, would exhibit higher and higher aspect ratios with increasing diameter of the bundled-bamboo structure, assuming constant aspect ratio of the bamboo-like structure. Then the single-crystalline pillars would fail more and more in a buckling mode. In this case of the bundled-bamboo structure, a larger cohesive strength of the grain boundaries is required for high aspect ratio grains to prevent buckling, which is intrinsically poor in HEAs^11^. If the deformation in each grain cannot be accommodated by its neighbours, it may lead to opening up voids and crack initiation along the boundaries^40^. This could explain why the large pillars eventually fail by intragranular fracture in contrast to the smaller ones where no fracture is observed.

The excellent thermal stability of the nanocrystalline HEA film could be attributed to their relatively low grain-boundary energy. Because grain interiors in the HEAs are highly disordered and far from a perfect crystal structure^11^,^41^, and the relative grain-boundary energy would be lowered than that of pure metals, such as pure W. Consequently, the driving force of grain-boundary migration in the HEA would be lower compared with pure W, leading to reduced structural coarsening. Other mechanisms that could contribute to the high stability of the HEA films are: at elevated temperatures the elements with higher diffusion rates may segregate to grain boundaries, decrease grain boundary-specific energy and stablize nanostructures against grain growth^26^; similar to the recently reported nanolaminated nickel^28^, the low-angle boundaries and high aspect ratios of grains in the columnar structure may reduce the mobility of grain boundaries as well as suppress recrystallization; the residual stresses in the HEA and W films in the annealing condition could also affect microstructural stability. Nonetheless, the refractory metals have very similar thermal expansion coefficients to the sapphire substrate at both room and high temperatures, so both the residual stresses of HEA and W would be significantly smaller than their yield strengths. Therefore, dislocation motion due to residual stress would not play a substantial role in grain growth compared with the other mechanisms.

**Figure 5:**
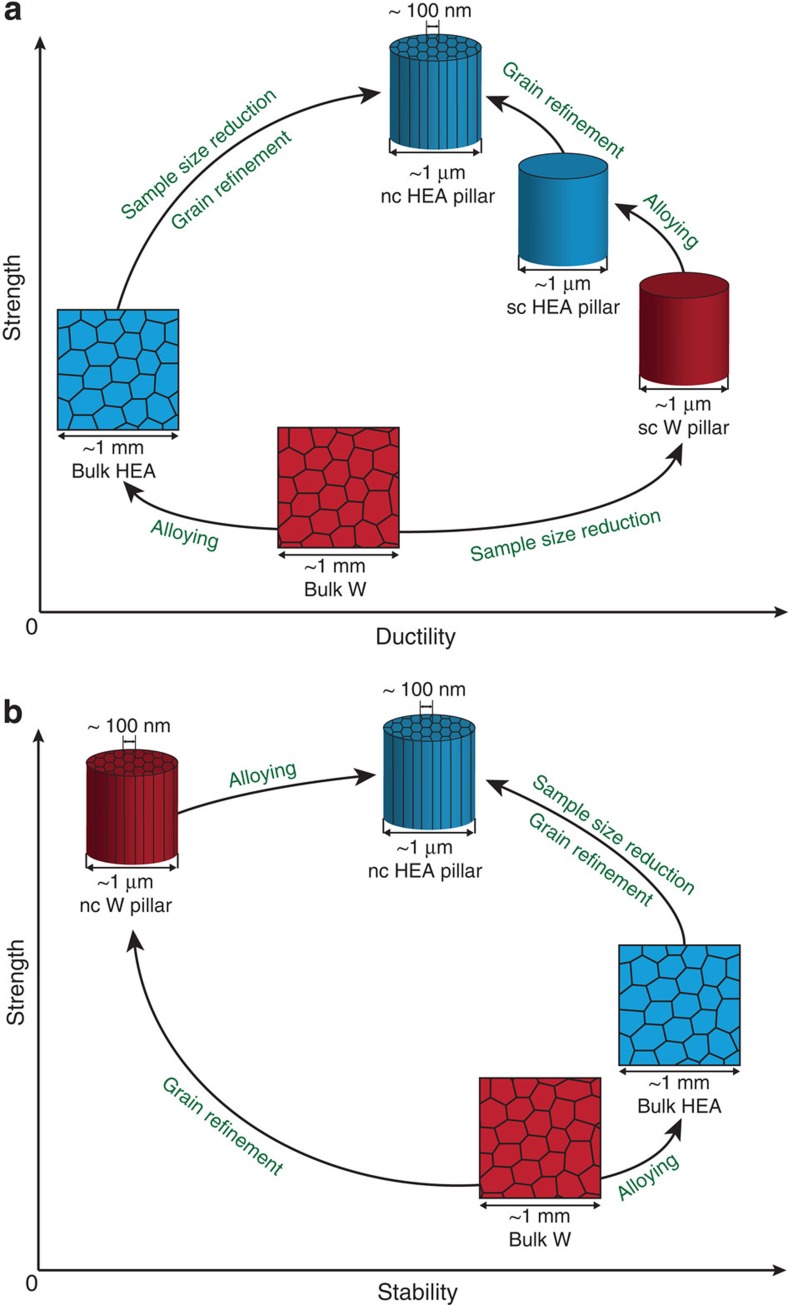
Schematic of strength-ductility and strength-stability synergies as comparing bulk coarse-grained W and HEA (NbMoTaW) to single-crystalline (sc) or nanocrystalline (nc) W and HEA. (**a**) The strength of a pure bulk W with coarse grains is increased by either alloying to a HEA at an expense of ductility (alloying effect) or by sample size reduction to a micrometre-sized single crystal with a benefit of being more ductile as well (sample size effect). The optimized strength-ductility combination can be achieved in a nc HEA micro-pillar with the benefits from sample size reduction, grain-boundary strengthening and solid-solution hardening. (**b**) The strength of a pure bulk W can be either significantly increased by grain refinement to a nc W but at a dramatic expense of thermal stability or increased by alloying to a bulk HEA with an improvement of stability. In a nanostructured HEA both extraordinary strength and excellent thermal stability can be achieved.

Figure 5 schematically illustrates how a strong, ductile and stable material is created by combining alloying effect and scaling laws. In contrast to the strength-ductility trade-off for a bulk coarse-grained W and HEA, both strength and ductility are significantly improved in nanocrystalline HEA micropillars, compared with a bulk HEA, benefiting from reduced sample size and grain refinement (Fig. 5a). With regards to strength-stability synergy (Fig. 5b), the drawback of thermal instability in nanocrystalline W can be overcome by alloying in nanocrystalline HEAs that also results a higher strength level.

Technologically, the fabrication and properties of this new class of small-dimension refractory HEAs are interesting and attractive. Although co-sputtering technique has been suggested to produce HEA films in some earlier reports^42^,^43^, to our knowledge this work constitutes the first report of the formation of single-phase nanostructured refractory HEAs. Furthermore, the fabrication process for these thin films is fast and controllable: the alloy composition, film thickness and grain size can be tuned.

Toward application, although the HEA films and pillars contain heavy elements, they still offer the highest specific-yield-strength values (strength-to-weight ratios) approaching 1 MJ kg^−1^ and high Young's modulus (Supplementary Fig. 6), and on this basis they surpass not only bulk metals and alloys but also other metallic pillars (Supplementary Fig. 7). The high specific strength of the small-scale HEAs combined with good ductility and high Young's modulus may permit access to high toughness, stiffness, hardness and wear resistance in a very high-stress environment, relative to other engineering materials. Last but not least, because the nanostructured HEAs are thermally stable at elevated temperatures and their bulk forms can even access large stresses above 1,600 °C, they may have a great opportunity to serve as high-temperature materials. Although mechanical tests for small-scale HEAs at high temperatures are still needed to prove this, our initial results of the HEA films under the high-temperature, long-duration conditions promise that they are capable of heat resistance and may serve as diffusion barriers and electrical resistors.

Despite much work remains to optimize small-scale HEAs for applications, for example, the best alloying elements and optimized grain and specimen size combination, the extraordinary properties of small-scale HEAs reported here offer a strong motivation to pursue their development.

## Methods

### Sample preparation and characterization

Our NbMoTaW HEA films were deposited using d.c. magnetron co-sputtering technique on (100) silicon substrates (coated with 50-nm SiO_2_ and 50-nm Si_3_N_4_ as diffusion barriers) or sapphire substrates (for annealing at 1,100 °C) at room temperature (Fig. 1b and Supplementary Fig. 1). The chamber base pressure was kept <10^−6^ mbar. During co-sputtering, the powers of the magnetrons were adjusted to obtain the equal arriving ratio of Nb, Mo, Ta and W, and the substrate was rotating as 30 rotations per minute in to homogenize alloy composition and film thickness. The IBAD method was also applied using a broad ion beam source (KRI KDC 40, beam energy of 1.2 keV, current of 5 mA and incidence angle of 35°) to decrease grain sizes, as compared with the Normal sputtering condition without ion gun. The film thickness is 3 μm and no difference was observed between the films deposited on silicon and sapphire substrates, in terms of the microstructure and mechanical properties. As a control, we also produced pure W films using the same conditions and parameters. The crystal orientations and elemental compositions of the films were characterized by EBSD and EDX, respectively, in a FEI Quanta 200 FEG SEM. The grain size and phase were determined by X-ray diffraction (Cu-Kα1 monochromatic radiation in a 2*θ* range from 10 to 100°).

From the obtained films, the pillar specimens were fabricated using a FIB system (Helios Nanolab 600i, FEI) with a coarse milling condition of 30 kV and 80 pA, and a final polishing condition of 5 kV and 24 pA. The FIB-milled pillars have diameters of ∼1 μm, 500, 200 and 100 nm, and aspect ratios of 2.5–5. The tapering angle is ∼2–4° and the top diameters were chosen to calculate engineering stresses.

### Mechanical testing

The microcompression tests were carried out in a nanoindenter using a diamond flat-punch tip. To eliminate strain-rate effects, we compressed all the pillars with a strain rate of 2 × 10^−3^ s^−1^ in the displacement control mode that was controlled by a feedback algorithm. It should be noted that a bigger tapering angle (>5°), a higher aspect ratio (>5) and the misalignment between the pillar top and flat punch could lead to very localized plastic deformation, buckling and bending, respectively. All the pillars were examined using scanning electron microscope (SEM) before and after compression tests, and those showing the above phenomena were eliminated to minimize these influences. The yield stress of pillars were measured as offset flow stress at 0.2% of strain. However, a large stress–strain scatter was usually observed in initial stage of plastic flow during compression, so the flow stresses at 5% of strain were used to compare the size effects.

### Heat treatment

To evaluate the thermal stability of the HEA and W films, we equilibrated the films with sapphire substrates at 1,100 °C in an argon atmosphere (the purity is ≥99,999, PanGas AG, Switzerland) for 3 days (heating and cooling rates are 100 °C h^−1^). Pre- and post-annealing films and pillar strengths were characterized, measured and compared.

## Additional information

**How to cite this article:** Zou, Y. *et al*. Ultrastrong ductile and stable high-entropy alloys at small scales. *Nat. Commun.* 6:7748 doi: 10.1038/ncomms8748 (2015).

## Supplementary Material

Supplementary Information Supplementary Figures 1-7
