# Supplementary material for: Ultrastrong ductile and stable high-entropy alloys at small scales
Source: Nat Commun. 2015 Jul 10;6:7748. doi: 10.1038/ncomms8748 (PMC4510962; doi:10.1038/ncomms8748)
Supplement: Supplementary Information — Supplementary Figures 1-7 [file ncomms8748-s1.pdf]

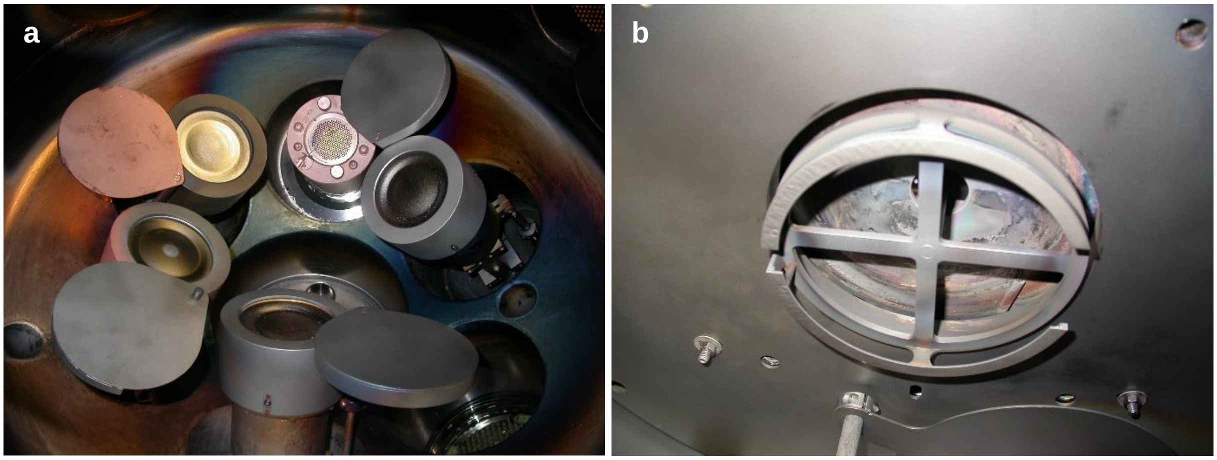

Supplementary Figure 1. The pictures of the magnetron co-sputtering system equipped with four targets arranged in a symmetry. This setup is used to synthesize the multi-component-alloy thin films: a, targets and the ion gun; b. the substrate holder.

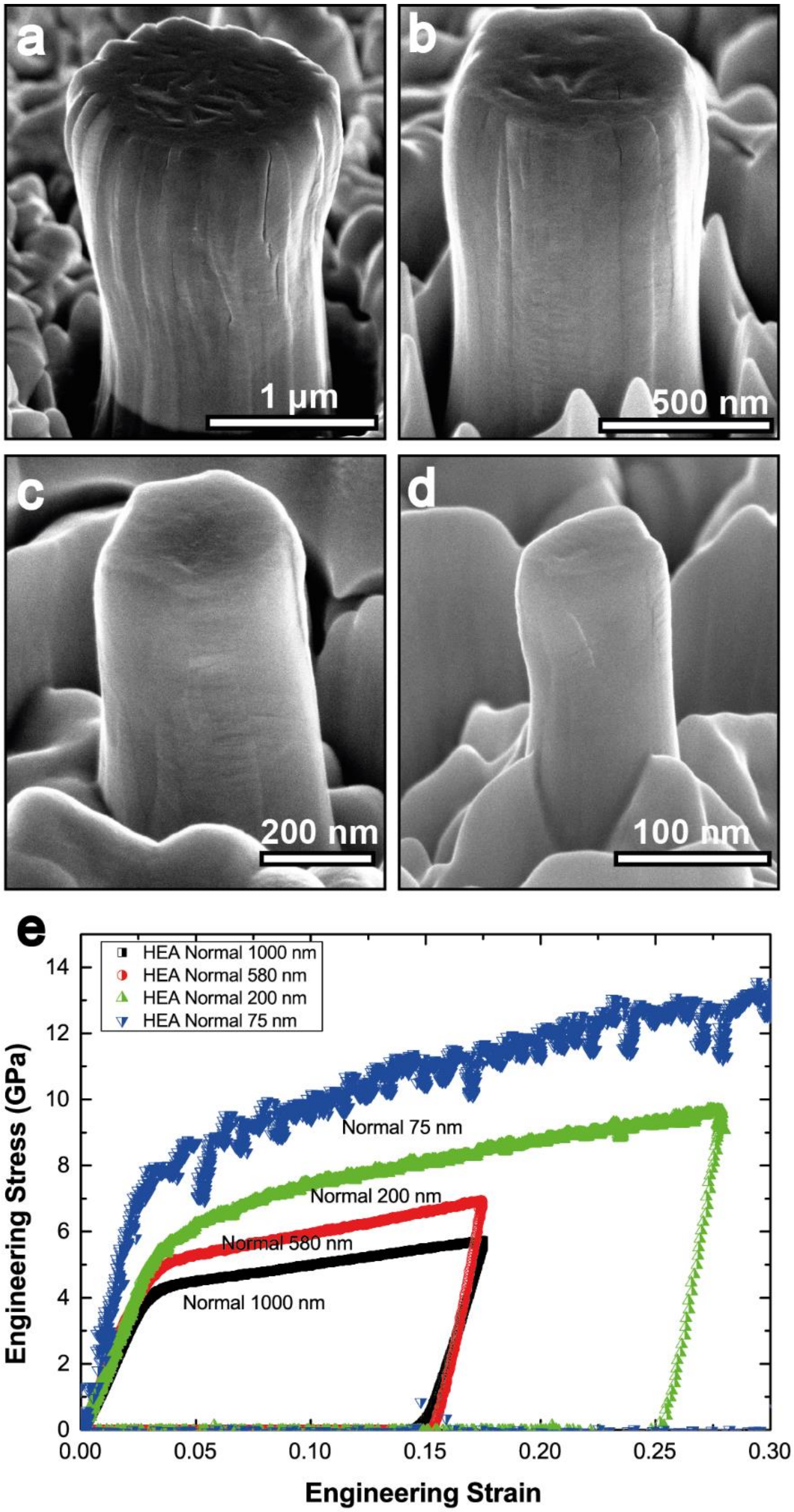

Supplementary Figure 2. a-d, SEM images of typical as-deformed HEA pillars (Normal) with the diameter (D) ranging from approximately 1 μm to 100 nm. e, Representative stress-strain curves of the HEA pillars, showing a size-dependent strength.

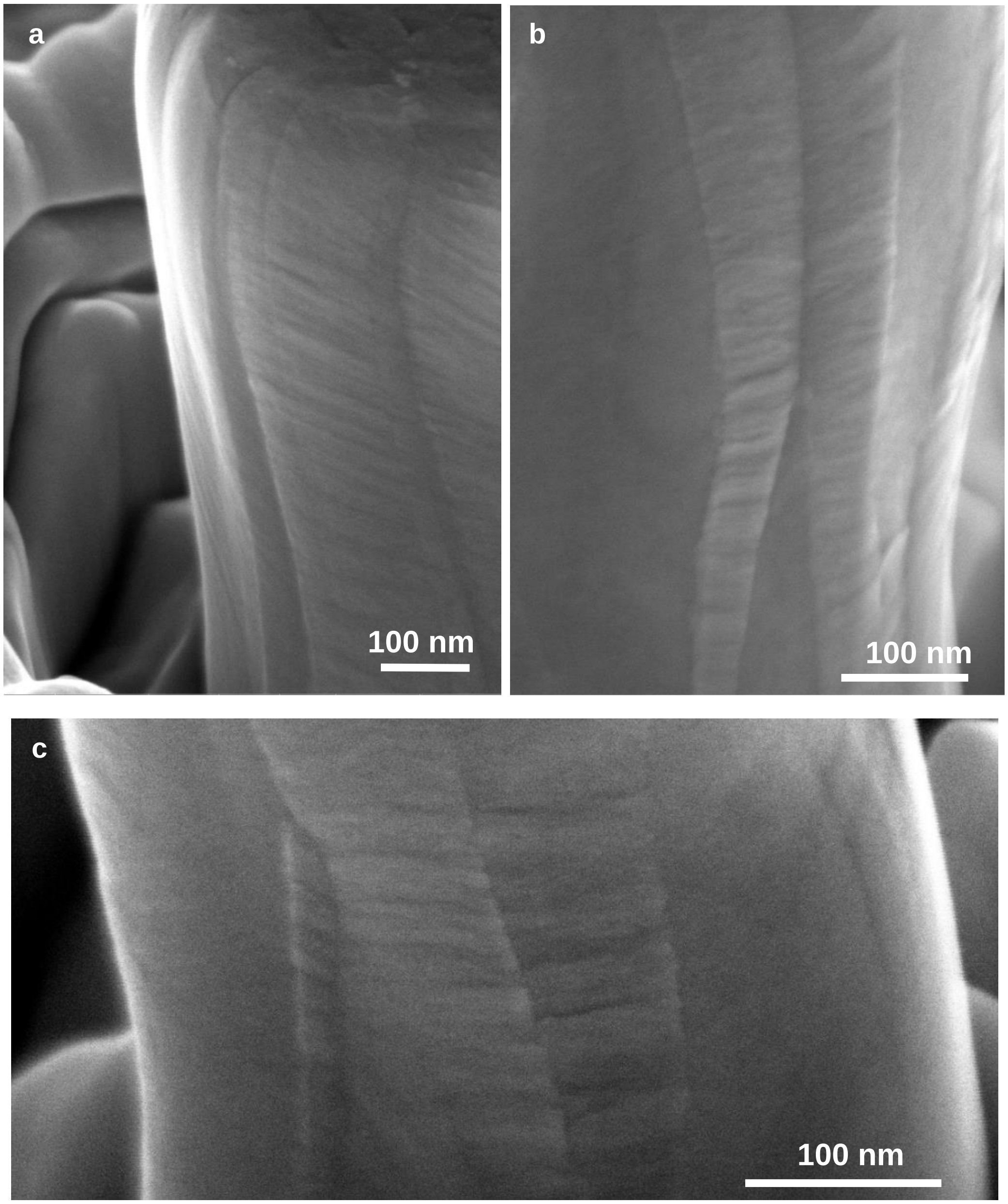

Supplementary Figure 3. High-resolution SEM images of typical as-deformed columnar-grain HEA pillar ([011] orientation with the diameter of ~500 nm)

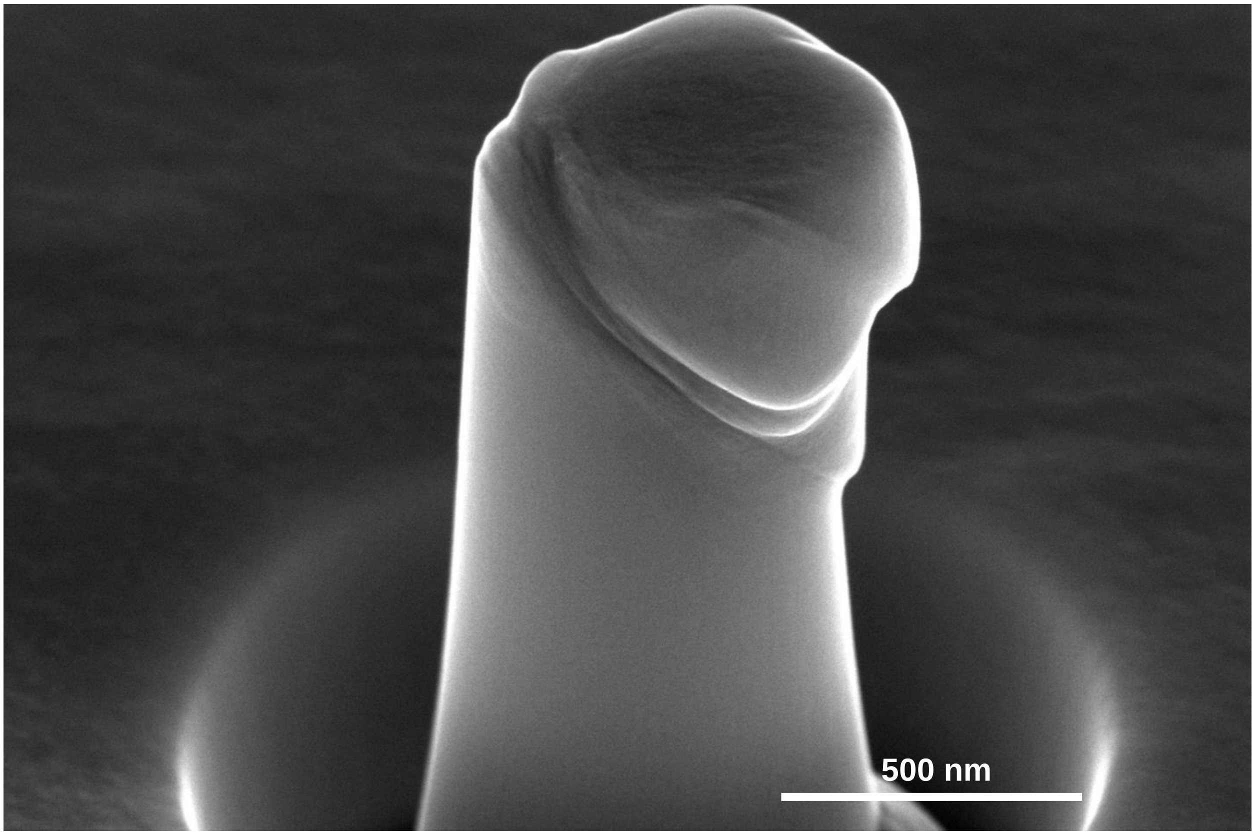

Supplementary Figure 4. SEM image of a typical as-deformed single-crystal HEA pillar ([011] orientation with the diameter of ~500 nm)

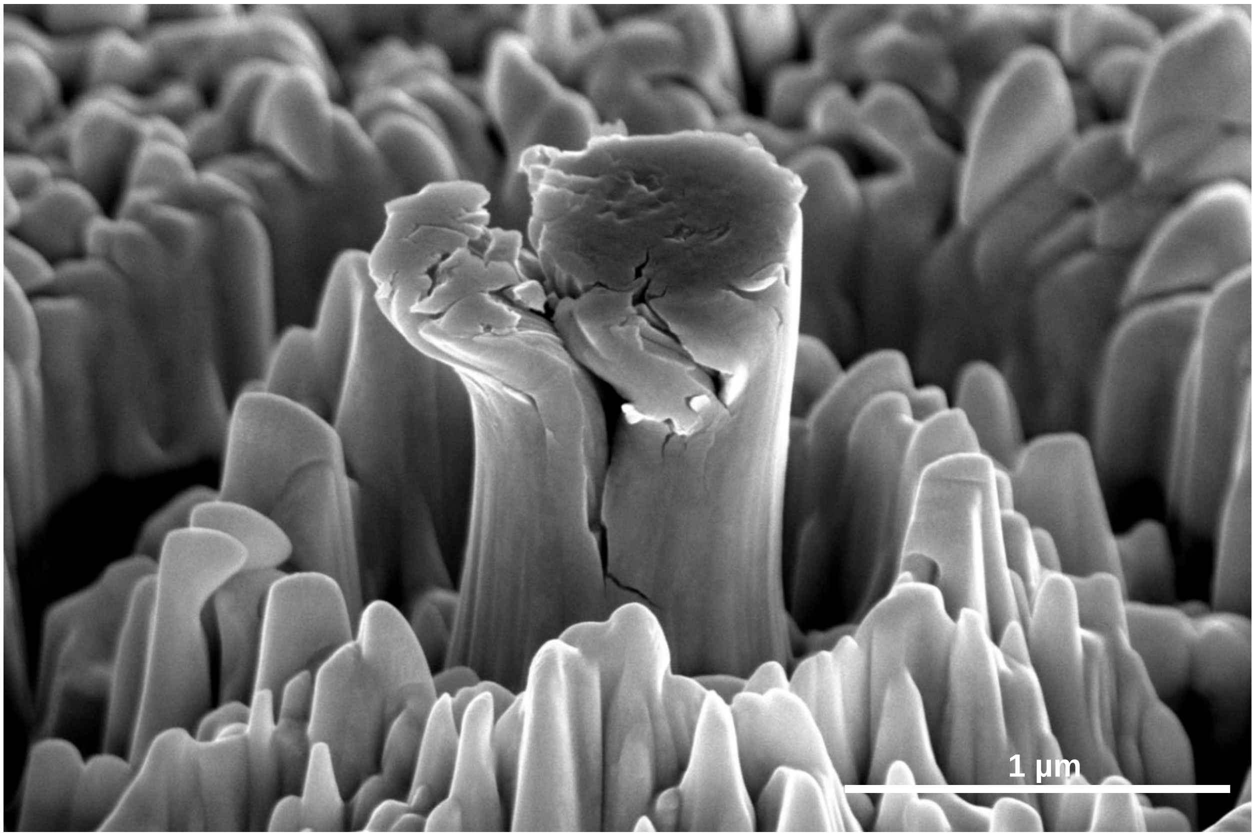

Supplementary Figure 5. SEM image of a typical as-deformed IBAD W pillar ([011] orientation with a diameter of ~700 nm), showing cracks propogage along the loading direction and indicating a brittle fracture behavior.

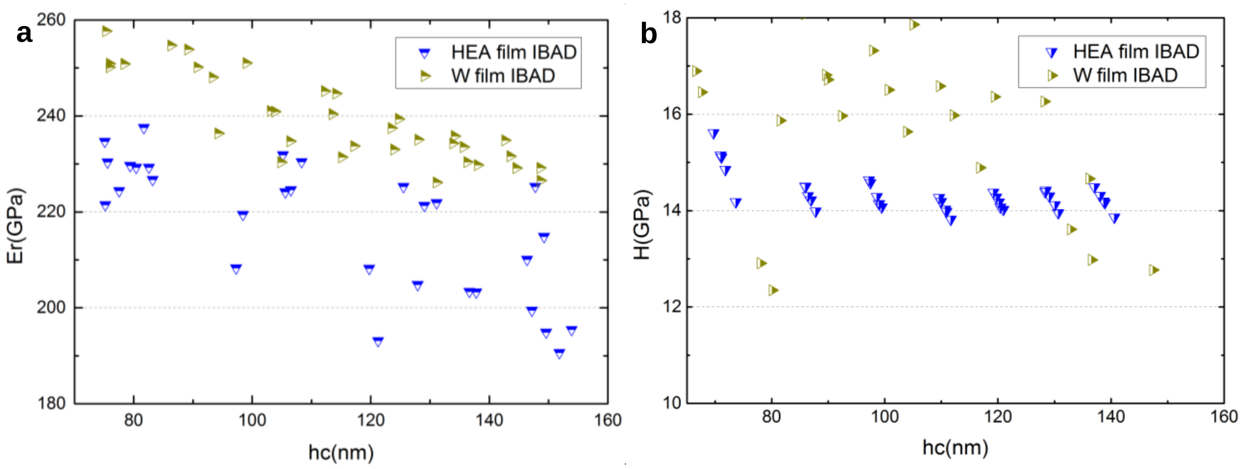

Supplementary Figure 6. Reduced modulus and hardness of the HEA and W films measured using nanoindenter with a Bekovich tip. The Young's moduls of the specimen can be calculated using the relation of

$\frac{1}{E_r} = \frac{1-\upsilon_i^2}{E_i} + \frac{1-\upsilon_s^2}{E_s}$. In our case, the specimen' modulus E_s is nearly the same as reduced modulus E_r. It should be note that W films have higher surface roughness than the HEA films and the roughness could also influence slightly on the measured modulus and hardness.

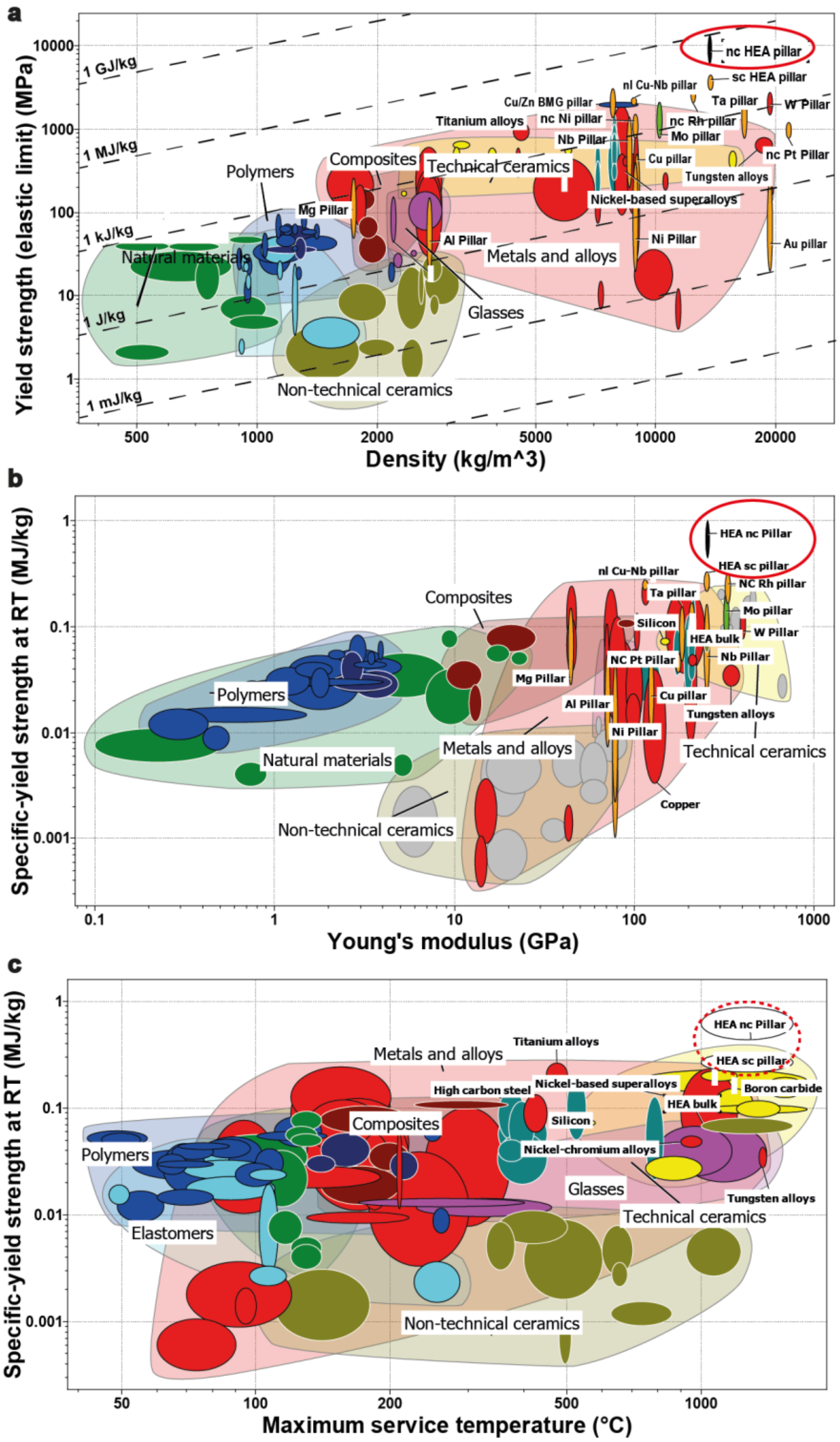

Supplementary Figure 7. Comparison of small-scale HEAs (single crystalline (sc) and nanostructured (nc) in this study) with various bulk materials and metallic pillars. Ashby maps of (a) yield strength vs. density, (b) specific-yield strength vs. Young's modulus and (c) specific-yield strength at room temperature vs. maximum service temperature (the service temperatures of the HEA pillars are assumed

from the compression tests of the bulk specimen). The yield strengths for bulk specimens are based on tensile tests and those for the pillars are obtained from micro-compression tests (The data base for bulk materials is according to Granta Design Limited CES EduPack 2014)
